# Impact of dysautonomic symptom burden on the quality of life in Neuromyelitis optica spectrum disorder patients

**DOI:** 10.1186/s12883-023-03162-1

**Published:** 2023-03-20

**Authors:** Lili Yang, Wenjing Li, Yan Xie, Shuai Ma, Xiaobo Zhou, Xinyue Huang, Song Tan

**Affiliations:** 1 Department of Neurology, Sichuan Academy of Medical Sciences & Sichuan Provincial People’s Hospital, University of Electronic Science and Technology of China, 32 West Second Section of First Ring Road, Chengdu, 610072 China; 2 Department of Psychosomatic, Sichuan Academy of Medical Sciences & Sichuan Provincial People’s Hospital, University of Electronic Science and Technology of China, Chengdu, 611731 China; 3 Sichuan Provincial Key Laboratory for Human Disease Gene Study, Chengdu, China; 4 Chinese Academy of Sciences Sichuan Translational Medicine Research Hospital, Chengdu, Sichuan China

**Keywords:** Neuromyelitis optica, Dysautonomic symptom burden, Autonomic nervous system, COMPASS-31, Quality of life

## Abstract

**Background:**

This study aimed to investigate the clinical risk factors of dysautonomic symptom burden in neuromyelitis optica spectrum disorder (NMOSD) and its impact on patients’ quality of life.

**Methods:**

A total of 63 NMOSD patients and healthy controls were enrolled. All participants completed the Composite Autonomic Symptom Score 31 (COMPASS-31) to screen for symptoms of autonomic dysfunction. A comprehensive clinical evaluation was performed on NMOSD patients, such as disease characteristics and composite evaluations of life status, including quality of life, anxiety/depression, sleep, and fatigue. Correlated factors of dysautonomic symptoms and quality of life were analyzed.

**Results:**

The score of COMPASS-31 in the NMOSD group was 17.2 ± 10.3, significantly higher than that in healthy controls (*P* = 0.002). In NMOSD patients, the higher COMPASS-31 score was correlated with more attacks (*r* = 0.49, *P* < 0.001), longer disease duration (*r* = 0.52, *P* < 0.001), severer disability (*r *= 0.50, *P* < 0.001), more thoracic cord lesions (*r* = 0.29, *P* = 0.02), more total spinal cord lesions (*r* = 0.35, *P* = 0.005), severer anxiety (*r* = 0.55, *P* < 0.001), severer depression (*r* = 0.48, *P* < 0.001), severer sleep disturbances (*r* = 0.59, *P* < 0.001), and severer fatigue (*r* = 0.56, *P* < 0.001). The disability, total spinal cord lesions, and fatigue were revealed to be independently associated factors. Further analysis revealed that the COMPASS-31 score was independently correlated with scores of all the domains of patients’ quality of life scale (*P* < 0.05).

**Conclusions:**

Dysautonomic symptom burden is correlated with decreased quality of life and certain clinical characteristics such as disability, the burden of spinal cord lesions, and fatigue in NMOSD patients. Investigation and proper management of autonomic dysfunction may help to improve the quality of life in patients with NMOSD.

**Supplementary Information:**

The online version contains supplementary material available at 10.1186/s12883-023-03162-1.

## Background

Neuromyelitis optica spectrum disorder (NMOSD), an idiopathic inflammatory central nervous system (CNS) disorder, is characterized by the presence of an antibody for the water channel aquaporin-4 (AQP4) and clinical characteristics, which are distinguished from multiple sclerosis (MS) [1]. The incidence of NMOSD is estimated at 0.57–4.52 per 100,000, with a high female-to-male ratio (3–9:1) [2, 3]. The incidence in the Chinese population is as high as 3.31/100,000 [4]. The most common clinical phenotypes are transverse myelitis (TM) and optic neuritis (ON) [1, 5].

Dysautonomic symptom burden is common in patients with neurological diseases, including MS [6, 7]. It has been found to seriously impact the patient’s quality of life [8–10] and is closely correlated with depression and fatigue [10, 11]. In recent years, research on autonomic dysfunction in NMOSD patients has been obtained. Two studies compared autonomic dysfunction between patients with NMOSD and those with MS [12, 13], finding a significant proportion of NMOSD patients suffered autonomic dysfunction and were more often symptomatic than MS patients in certain domains of dysautonomia. Another study suggested 74.1% of patients with NMOSD suffered dysautonomic symptoms and investigated the correlated clinical and MRI factors of dysautonomia [14]. However, current research on autonomic dysfunction in NMOSD has not been able to explore its correlation with patients' life status (depression, anxiety, sleep, fatigue), and the extent of its impact on patients’ quality of life.

Herein, we investigated dysautonomic symptom burden by using a well-recognized autonomic symptom questionnaire, the Composite Autonomic Symptom Score (COMPASS-31), in patients with NMOSD, as well as explored its impact on patient’s quality of life and its clinical correlates among demographic features, disease characteristics, and life status evaluations.

## Methods

### Participants

We prospectively enrolled patients with NMOSD, who visited our clinic from June 2021 to July 2022. NMOSD was diagnosed according to the 2015 International Panel for Neuromyelitis Optica diagnosis (IPND) criteria [1]. The exclusion criteria were as follows: (1) history of drug or alcohol abuse or other major clinical or psychiatric conditions, especially those that could result in autonomic dysfunction, such as parkinsonism, Sjogren's syndrome, amyloidosis, leprosy, polyneuropathy, diabetes, etc.; (2) receiving acute immunoregulatory treatment; (3) with cerebral lesions or severe visual impairments; (4) taking medications such as antihypertensives, anticholinergics, and antiarrhythmics, which may influence autonomic function; and (5) inability to complete all questionnaires with the assistance of neurologists. Demographic data and disease features were collected including gender, age, body mass index (BMI), the presence of antibodies associated with CNS demyelinating disease in serum (including AQP4-Ab, anti-myelin oligodendrocyte glycoprotein antibody (MOG-Ab), and anti-glial fibrillary acidic protein antibody (GFAP-Ab)), the number of clinical attacks, disease duration, the segments of spinal cord lesions (cervical, thoracic, lumbosacral and total), degree of disability, clinical phenotype, and current preventive therapy. Antibodies in serum were tested with a transfected cell-based assay [15]. The degree of disability was evaluated by two neurologists according to the Expanded Disability Status Scale (EDSS) score [16]. Severe visual impairment was referred to as a visual function subscore of 6 according to the EDSS score. The segments of spinal cord lesions were acquired from 3T spine magnetic resonance images.

Healthy controls (HCs) were recruited among individuals who attended the hospital for annual health check-ups, with no major clinical or psychiatric conditions. Demographic data and BMI regarding the HCs were collected. The Ethics Committee of Sichuan Provincial People’s Hospital approved the study. Recruited participants provided written informed consent before enrolling in the study.

### Composite Autonomic Symptom Score 31

All participants were requested to complete the COMPASS 31 questionnaire independently according to the actual situation. COMPASS-31 comprises 6 domains with 31 items (orthostatic intolerance 4 items, vasomotor 3 items, secretomotor 4 items, gastrointestinal 12 items, bladder 3 items, and pupillomotor 5 items) and provides the minimal weighted total score equals 0 and the maximum weighted total score equals 100 [17]. The higher the score, the more severe the dysautonomic symptoms.

### Clinical composite evaluation of living status in NMOSD patients

All enrolled NMOSD patients were requested to fulfill a composite evaluation of living status, including anxiety, depression, sleep quality, and fatigue. The Hospital Anxiety and Depression Scale (HADS) used in this study was developed to identify cases of anxiety disorders and depression among patients in nonpsychiatric hospital clinics [18]. It is divided into an anxiety subscale (HADS-A) and a depression subscale (HADS-D), both containing seven intermingled items. The score of the HADS-A and HADS-D both ranges from 0 to 21, and higher scores indicate more severe anxiety/depression. The sleep quality of patients was assessed through the Pittsburgh Sleep Quality Index (PSQI) [19]. The global PSQI score ranges from 0 to 21, with higher scores indicating worse sleep quality. Fatigue was evaluated with the Fatigue Severity Scale (FSS), which was a self-report instrument to evaluate patients' perceptions of the functional limitations caused by fatigue within the last week [20]. Possible global scores range from 7 to 63, where higher scores indicate more severe fatigue.

### Quality of life evaluation of NMOSD patients

All NMOSD patients completed the evaluation of their quality of life via the 36-item short-form health survey (SF-36). SF-36 evaluates 8 dimensions: physical functioning (10 items), physical role fulfillment (4 items), bodily pain (2 items), general health (5 items), vitality (4 items), social functioning (2 items), emotional role fulfillment (3 items), and mental health (5 items) [21]. The scale’s total possible score is 145, with a higher score reflecting better quality of life.

All the scales used in the present study have been utilized in previous NMOSD studies [13, 14, 22–24]. All the Chinese versions of these scales have been validated previously [25–29]. A psychologist administered the HADS questionnaires; the other questionnaires were completed by the participants themselves in the presence of a neurologist, who assisted the participants in reading and understanding the items.

### Statistical analysis

All statistical analyses were carried out using the statistical software GraphPad Prism (version 8, San Diego, CA).

To compare the demographic characteristics between the NMOSD and HC groups, Fisher’s exact test was used in the gender ratio analysis, and the Mann–Whitney U test was used in the comparisons of age, BMI, COMPASS-31 score, and its subscores.

To analyze the related factors of dysautonomic symptoms in NMOSD, Mann–Whitney and Kruskal–Wallis tests were used to determine whether COMPASS-31 scores/subscores differed among groups defined by gender, AQP4 seropositivity or seronegativity, clinical phenotype, and current preventive therapy. Spearman’s ranked correlation analysis was used to explore the relationships between the COMPASS-31 score/subscores and the independent variables, including age, BMI, number of attacks, disease duration, EDSS, segments of spinal cord lesions (cervical, thoracic, lumbosacral, total separately), HADS-A, HADS-D, PSQI, and FSS score. Multiple linear regression was used to further assess the independent factor of the COMPASS-31 score/subscore (checking the normality of residuals). Age, gender, BMI, clinical phenotype, number of attacks, disease duration, current therapy, EDSS, segments of total spinal cord lesions, HADS, PSQI, and FSS score were included as possible independent variables for the multiple linear regression model. *P* < 0.05 was considered statistically significant.

To analyze the influence of dysautonomic symptom burden on patients’ quality of life, another multiple linear regression model was established to assess the independent contributor of different domains of SF-36 score in NMOSD patients (checking the normality of residuals). Age, gender, BMI, clinical phenotype, number of attacks, disease duration, current therapy, EDSS, COMPASS-31, HADS, PSQI, and FSS score were included as possible independent variables for this multiple linear regression model. *P* < 0.05 was considered statistically significant.

## Results

### Demographic data of NMOSD patients and HCs

A total of 63 NMOSD patients and 63 HCs were enrolled, with no significant difference in age or gender (seen in Table 1).

**Table 1:** Demographic, clinical characteristics and composite evaluation in NMOSD and HC

	NMOSD (*n* = 63)	HC (*n* = 63)	*P* value
Age (years), mean [SD] (range)	41 [13.8] (18–69)	37.8 [12.5] (20–60)	0.50
Sex (F), N (%)	50 (79.4%)	47 (74.6%)	0.67
BMI, mean [SD] (range)	22.7 [4.0] (12.6–35.4)	22.7 [4.1] (17.1–39.0)	0.59
Antibody in serum			
AQP4 seropositivity, N(%)	55 (87.3%)		
MOG seropositivity, N(%)	4 (6.3%)		
Negative	4 (6.3%)		
Number of attacks, mean [SD] (range)	3.7 [3.9] (1–21)		
Disease duration (year), mean [SD] (range)	4.2 [4.3] (0.25–20)		
EDSS, mean [SD] (range)	3.1 [1.9] (0–7.5)		
Clinical phenotype, N(%)			
ON	15 (23.8%)		
TM	22 (34.9%)		
ON + TM	26 (41.3%)		
Segments of MR lesions, mean [SD] (range)			
Cervical cord lesions	2.2 [2.2] (0–7)		
Thoracic cord lesions	2.2 [3.4] (0–12)		
Total number of spinal cord lesion	4.3 [4.3] (0–17)		
Therapy, N(%)			
MMF	37 (58.7%)		
AZA	5 (7.9%)		
RTX	17 (30.0%)		
periodic IVIG	1 (1.6%)		
only low-dose prednisolone	3 (4.8%)		
COMPASS-31, mean [SD] (range)	17.2 [10.3] (1–43)	11.6 [7.3] (0–32)	**0.002**
Orthostatic intolerance	1.8 [2.3] (0–10)	1.1 [1.9] (0–6)	**0.003**
Vasomotor	0.8 [1.7] (0–6)	0.2 [0.8] (0–5)	**0.007**
Secretomotor	2.1 [1.7] (0–6)	1.3 [1.4] (0–6)	**0.006**
Gastrointestinal	6.2 [4.0] (0–15)	5.2 [3.8] (0–16)	0.18
Bladder	1.4 [1.7] (0–6)	0.4 [0.9] (0–4)	** < 0.001**
Pupillomotor	4.7 [3.5] (0–14)	3.4 [2.9] (0–10)	**0.04**

*F* female, *M* male, *BMI* Body Mass Index, *AQP4-IgG* IgG autoantibodies to aquaporin 4, *EDSS* Expanded Disability Status Scale, *ON* optica neuritis, *TM* transverse myelitis, *MMF* mycophenolate mofetil, *AZA* azathioprine, *RTX* rituximab, *IVIG* intravenous immunoglobulin, *COMPASS-31* Composite Autonomic Symptom Score 31

The disease characteristics of NMOSD patients were also summarized in Table 1. The distributions of the clinical phenotype were as follows: 15 patients had only ON (23.8%), 22 patients had only TM (34.9%), and 30 patients had both ON and TM (41.3%). Four patients in the ON + TM subgroup also experienced area postrema syndrome. The segments of spinal cord lesions were collected, while none of the patients has a lumbosacral spinal cord involvement.

Regarding the distribution of current preventive therapy, 42 patients (66.7%) used immunosuppressants such as mycophenolate mofetil (MMF, 37 patients) or azathioprine (AZA, 5 patients); 17 patients (30.0%) received rituximab (RTX); and the remaining 4 patients used other therapies, such as periodic intravenous immunoglobulin (1 patient) or only low-dose prednisolone (3 patients).

### The comparison of COMPASS score/subscore between NMOSD patients and HCs

The COMPASS-31 score in NMOSD patients was 17.2 ± 10.3, significantly higher than that in HCs (*P* = 0.002). For the six subdomains of COMPASS-31, the NMOSD patients had significantly higher scores than HCs in orthostatic intolerance (*P* = 0.003), vasomotor (*P* = 0.007), secretomotor (*P* = 0.006), bladder (*P* < 0.001), and pupillomotor (*P* = 0.04) scores. The statistical results could be seen in Table 1.

We also analyzed the frequency of patients with general dysautonomic symptoms (COMPASS-31 score > 0), as well as the frequency of those with dysfunction in each dimension (the corresponding subscore > 0). The results showed that all 63 NMOSD patients suffered dysautonomic symptoms, the number and frequency of NMOSD patients suffering dysfunction in each dimension were as follows: 40 patients (63.5%) with orthostatic intolerance, 42 (66.7%) with vasomotor symptoms, 57 (90.5%) with secretomotor symptoms, 60 (95.2%) with gastrointestinal symptoms, 49 (77.8%) with bladder symptoms, and 58 (92.1%) with pupillomotor symptoms.

### Clinical factors associated with the COMPASS-31 total score/subscore in NMOSD patients

**Fig. 1:**
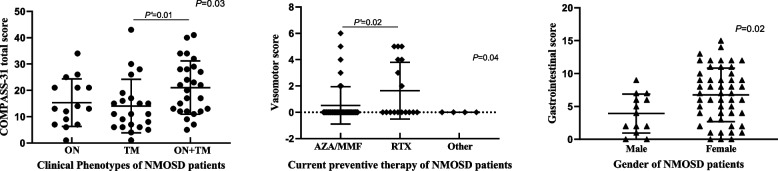
The comparisons of COMPASS-31 score/subscore among NMOSD subgroups, only displaying the significantly different results (*P* < 0.05). The detailed results of the comparisons of COMPASS-31 score/subscore among NMOSD subgroups distributed by gender, AQP4 seropositivity or seronegativity, clinical phenotype, and current preventive therapy could be seen in Supplementary Table S1. COMPASS-31, Composite Autonomic Symptom Score 31. ON, optica neuritis; TM, transverse myelitis; MMF, mycophenolate mofetil; AZA, azathioprine; RTX, rituximab

We further analyzed clinical factors associated with dysautonomic symptoms in NMOSD patients, to reveal the relevant factors and intervention targets. Firstly, we analyzed the difference in COMPASS-31 total scores/subscores among NMOSD subgroups distributed by gender, AQP4 seropositivity or seronegativity, clinical phenotype, and current preventive therapy, considering each of these independent variables separately. We found that the COMPASS-31 total scores were significantly different among patients with different clinical phenotypes (*P* = 0.03) (Fig. 1, Supplementary Table S1). Regarding subscores, the scores of vasomotor symptoms were significantly different in patients with different preventive therapy (*P* = 0.04). Meanwhile, female patients showed higher scores of gastrointestinal symptoms than male patients (*P* = 0.02). There were no significant differences in the COMPASS-31 total score and subscore among the other NMOSD subgroups.

**Fig. 2:**
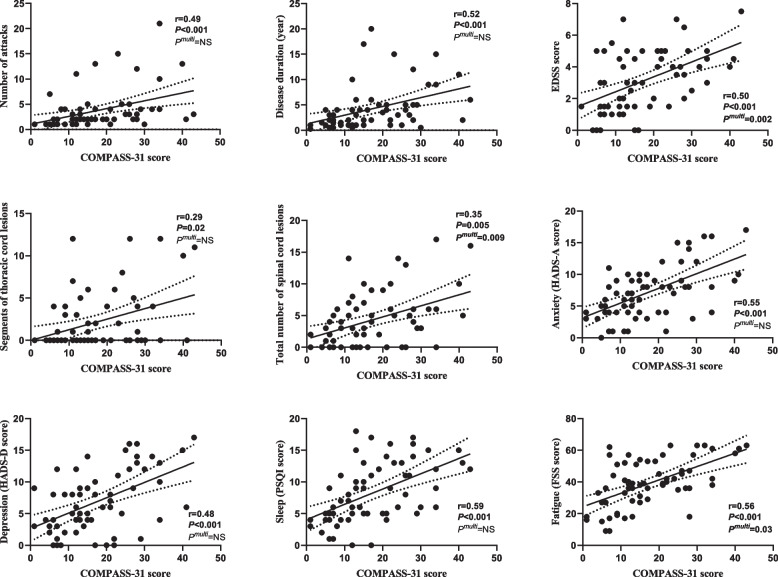
Correlation and multiple linear regression analysis between COMPASS-31 score and several independent variables. The *r* values and *P* values are labeled. A multiple linear regression model was used to distinguish any factors that were independently related to the COMPASS-31 score, and P^multi^ indicates the statistical result. A value of *P* < 0.05 was considered significant. * Asterisks mark the variables that are significantly correlated with COMPASS-31 scores in the correlation analysis; COMPASS-31 scores were correlated with the number of attacks, disease duration, EDSS, segments of spinal cord lesions (thoracic, total), HADS-A, HADS-D, PSQI, and FSS. # This symbol indicates that the variable was independently correlated with the COMPASS-31 score in the multiple linear regression model. EDSS, segments of total spinal cord lesions, and FSS were the independent correlated factor. COMPASS-31, Composite Autonomic Symptom Score 31; EDSS, Expanded Disability Status Scale; HADS-A, Hospital Anxiety and Depression Scale–Anxiety; HADS-D, Hospital Anxiety and Depression Scale–Depression; PSQI, Pittsburgh Sleep Quality Index; FSS, Fatigue Severity Scale

Furthermore, we performed correlation analyses between COMPASS-31 scores/subscores and other quantitative variables separately. The results demonstrated that higher COMPASS-31 scores were correlated with more attacks (*r* = 0.49, *P* < 0.001), longer disease duration (*r* = 0.52, *P* < 0.001), higher EDSS score (*r* = 0.50, *P* < 0.001), more thoracic cord lesions (*r* = 0.29, *P* = 0.02), more total spinal cord lesions (*r* = 0.35, *P* = 0.005), higher HADS-A (*r* = 0.55, *P* < 0.001), higher HADS-D (*r* = 0.48, *P* < 0.001), higher PSQI (*r* = 0.59, *P* < 0.001), and higher FSS (*r* = 0.56, *P* < 0.001) in NMOSD patients (displayed in Fig. 2).

Regarding the subscore of COMPASS-31, only the score of vasomotor symptoms was moderately correlated with HADS-A (*r* = 0.35, *P* = 0.004), while other correlations were mildly correlated with limited significance. The detailed results could be seen in Supplementary table S2.

In the multivariable linear regression analysis model, we found the independently associated variables of the COMPASS-31 score were the EDSS score (*P* = 0.002), the total number of spinal cord lesions (*P* = 0.009), and the FSS score (*P* = 0.031) (seen in Fig. 2). There were no independently associated variables of COMPASS-31 subscores. The detailed statistical results could be seen in Supplementary table S3.

### The influencing factors of the quality of life in NMOSD patients

Through a multivariable linear regression model, which included comprehensive underlying factors affecting the patient’s quality of life, we found that the COMPASS-31 score was the independently correlated factor of all the domains of SF-36 (*P* < 0.05). BMI was also found to be the independently correlated factor of the score of general health in SF-36 (*P* = 0.030). The detailed statistical data could be seen in Table 2.

**Table 2:** Multivariable linear regression of the SF-36 in NMOSD patients

Variables	B	S.E	95%CI	*P*
Physical Functioning				
COMPASS-31	-1.88	0.44	-2.76 to -0.99	** < 0.001**
Role Physical				
COMPASS-31	-1.95	0.65	-3.27 to -0.64	**0.004**
Bodily Pain				
COMPASS-31	-1.12	0.29	-1.71 to -0.54	** < 0.001**
General Health				
COMPASS-31	-1.18	0.27	-1.72 to -0.63	** < 0.001**
BMI	-1.28	0.57	-2.43 to -0.13	**0.030**
Vitality				
COMPASS-31	-1.15	0.35	-1.85 to -0.46	**0.002**
Social Functioning				
COMPASS-31	-1.43	0.38	-2.19 to -0.67	** < 0.001**
Role Emotional				
COMPASS-31	-2.60	0.66	-3.94 to -1.27	** < 0.001**
Mental Health				
COMPASS-31	-0.58	0.19	-0.96 to -0.21	**0.003**
Reported Health Transition				
COMPASS-31	1.14	0.53	0.08 to 2.20	**0.035**

*SF-36* the 36-item short-form health survey, *BMI* Body Mass Index, *COMPASS-31* Composite Autonomic Symptom Score 31

## Discussion

Our study found that NMOSD patients reported more severe dysautonomic symptoms than the HCs with age and gender matched. We further revealed the associated factors of dysautonomic symptoms in NMOSD patients. Among the correlated factors, the EDSS score, the total number of spinal cord lesions, and fatigue were independent risk factors. Further analysis showed that dysautonomic symptom burden was an independent influencing factor in all domains of scale on patients’ quality of life in NMOSD. To our knowledge, it’s the first investigation of the impacts of dysautonomic symptom burden on patients’ quality of life and the associations of dysautonomic symptoms with the life status evaluations in NMOSD.

The autonomic function is commonly assessed in two ways, patient-reported symptom studies (often using different questionnaires) and assessment of autonomic function/dysfunction in the laboratory [30]. Laboratory evaluation, although more objective, is time-consuming since it requires multiple tests regarding different domains of autonomic function. Moreover, it may only be positive in patients with severe autonomic dysfunction. For example, a previous study combined the self-reported questionnaires and laboratory measurements in NMOSD patients showed that all patients had self-reported dysautonomic symptoms (20 patients, COMPASS > 0), but only 11 (55%) were positive for tests [13]. The patients with negative laboratory tests do endure dysautonomic symptoms subjectively, which would impact their health status. These subjective feelings should not be directly ignored when investigating the impact of dysautonomic symptom burden on the quality of life in NMOSD patients. Previous MS studies have proved that patients with laboratory-confirmed autonomic dysfunction score higher on certain domains of the COMPASS-31 scale [30, 31], suggesting that the quantified COMPASS-31 score was in good agreement with laboratory tests. Taking into account all these considerations, we used the COMPASS-31 scale to analyze the autonomic function in NMOSD patients, to comprehensively and sensitively identify the patients’ dysautonomic symptom burden.

Autonomic dysfunction is common in the general population, especially in the elderly [11], since hormonal changes and oxidative stress also participate in the progress of autonomic dysfunction [32]. However, the previous NMOSD study of autonomic dysfunction did not enroll healthy participants with age and gender matched as controls [13]. Our study had filled this blank. Through comparison with age-gender-matched HCs, patients with NMOSD were found to have significantly higher scores than HCs in multiple domains of dysautonomic symptoms, except gastrointestinal symptoms. This finding suggested that NMOSD patients were more likely to suffer orthostatic intolerance, vasomotor symptoms, secretomotor symptoms, bladder symptoms, and pupillomotor symptoms than the general population, while the gastrointestinal symptoms in NMOSD patients might not be disease-related.

We further analyzed clinical factors associated with dysautonomic symptoms and found that the degree of disability (EDSS score), spinal cord lesion burden, and fatigue were independent risk factors for dysautonomic symptoms in patients with NMOSD. Our findings were consistent with the previous studies in NMOSD [14] and earlier studies in MS [30, 33] regarding the association of dysautonomic symptoms with disability and spinal cord lesion burden. Our stratified analysis in NMOSD patients with myelitis also showed that the more severe sequela after myelitis (represented by the sum scores of pyramidal, sensory, bowel & bladder, and walking subdomains in EDSS) was correlated with more severe autonomic dysfunction in NMOSD patients. It can be explained by the anatomical features of the autonomic nervous system, where the preganglionic cells of the sympathetic nervous system are located between the thoracic and upper lumbar segments of the spinal cord [14]. However, autonomic dysfunction may not only be a consequence of disease progression, but it may also be a contributor. Previous studies suggested that the autonomic nervous system and the immune system interacted on several levels [34]. The parasympathetic nervous system may play an important role in alerting the CNS to the presence of inflammation through the "cholinergic anti-inflammatory pathway" [30]. It is speculated that this pathway suppresses inflammation and immune responses by integrating signals from the immune system and the nervous system [7, 35]. Another branch of the ANS, the sympathetic system, was also found to modulate the CNS inflammatory response in MS through laboratory and genetic studies [36, 37]. Whether the autonomic dysfunction in NMOSD was a promoting factor in the disease progression needed to be further researched.

The close relationship between dysautonomic symptoms and fatigue in NMOSD patients was revealed for the first time in this study. Fatigue is extremely common among NMOSD patients and can significantly influence patients’ quality of life, according to recent clinical findings [38–40]. As a complex and multifactorial problem, the underlying pathophysiology of fatigue is unclear. A close relationship between dysautonomic symptoms and fatigue had also been found in MS [10], and some hypothesized that fatigue may be attributed to the orthostatic intolerance of MS patients [41, 42]. Researchers also demonstrated that MS patients with fatigue had adrenergic hyporesponsive [41], which was not found in MS patients without fatigue or normal controls. Our findings suggested that autonomic dysfunction might play an important role in the underlying mechanism of NMOSD fatigue.

Further, through a multiple regression model integrating multifactorial variables, we were surprised to find that dysautonomic symptom burden was an independent influencing factor of all the domains of the quality of life assessed by SF-36. Dysautonomic symptoms have been demonstrated to cause physical and emotional discomfort, limit the activities of daily living and social participation, and ultimately affect the health-related quality of life [11]. Studies in many diseases provide overwhelming evidence for an association between the presence of autonomic symptoms and reduced quality of life [8, 10, 43]. In the study of NMOSD, earlier studies showed that bowel and bladder dysfunction was associated with quality of life or spinal cord atrophy [44, 45]. We confirmed the close relationship between the quality of life in NMOSD patients and the self-reported overall autonomic dysfunction. Our findings suggest that more attention should be paid to screening and managing the dysautonomic symptoms of NMOSD patients, which may be vital in improving patients’ quality of life.

### Limitation

This study has some limitations. First, this study was a cross-sectional observational study that only evaluated the influence of dysautonomic symptoms on quality of life, while further prospective studies are needed to judge the impact of dysautonomic symptoms on disease activity or prognosis. Second, the COMPASS 31 questionnaire requires memory data on various autonomic symptoms that reflect subjective experiences and feelings. The subjective nature of the assessment tools employed sacrificed certain accuracies. Third, this study was a single-center clinical study with a limited sample size, future studies with larger samples and stratified analyses in patients with different disease severity (for example, different degrees of disability or spinal cord injury) are needed to validate our findings and to reveal promising therapeutic targets for improving dysautonomic symptoms.

## Conclusion

The present study investigated self-perceived dysautonomic profiles by using COMPASS-31 in patients with NMOSD and found that the dysautonomic symptom burden in NMOSD patients was much more severe than HCs. Disability, burden of spinal cord lesions, and fatigue were independently correlated with dysautonomic symptoms in NMOSD patients. Moreover, the dysautonomic symptom burden was an independent influencing factor of the patients’ quality of life in NMOSD. These findings might help us identify patients with a high risk of autonomic dysfunction and suggest that the evaluation and management of autonomic dysfunction are of great significance for improving the quality of life in NMOSD patients.

## Supplementary Information

**Additional file 1: Supplementary Table S1.** The comparisons of COMPASS-31 score/subscore among NMOSD subgroups distributed by gender, serum AQP4-IgG positive or negative, clinical phenotype, and current preventive therapy. **Supplementary Table S2.** The correlation between COMPASS-31 score/subscore and clinical variables in NMOSD patients. **Supplementary Table S3.** Multivariable linear regression of the COMPASS-31 score in NMOSD patients.

## Data Availability

The data that support the findings of this study are available from the department of Neurology of Sichuan Provincial People’s Hospital but restrictions apply to the availability of these data, which were used under license for the current study, and so are not publicly available. Data are however available from the authors upon reasonable request and with permission of the research deputy of the department of Neurology of Sichuan Provincial People’s Hospital. Researchers could contact L.Y. (yanglili@med.uestc.edu.cn) if there is a need.

## Abbreviations

NMOSD: Neuromyelitis optica spectrum disorder
CNS: Central nervous system
AQP4: Aquaporin-4
MS: Multiple sclerosis
TM: Transverse myelitis
ON: Optic neuritis
COMPASS-31: Composite Autonomic Symptom Score 31
IPND: International Panel for Neuromyelitis Optica diagnosis
BMI: Body mass index
EDSS: Expanded Disability Status Scale
HCs: Healthy controls
HADS: Hospital Anxiety and Depression Scale
PSQI: Pittsburgh Sleep Quality Index
FSS: Fatigue Severity Scale
SF-36: The 36-item short-form health survey
MMF: Mycophenolate mofetil
AZA: Azathioprine
RTX: Rituximab
