# Supplementary material for: Impact of dysautonomic symptom burden on the quality of life in Neuromyelitis optica spectrum disorder patients
Source: BMC Neurol. 2023 Mar 20;23:112. doi: 10.1186/s12883-023-03162-1 (PMC10026430; doi:10.1186/s12883-023-03162-1)
Supplement: Supplementary file 1 — Additional file 1: Supplementary Table S1. The comparisons of COMPASS-31 score/subscore among NMOSD subgroups distributed by gender, serum AQP4-IgG positive or negative, clinical phenotype, and current preventive therapy. Supplementary Table S2. The correlation between COMPASS-31 score/subscore and clinical variables in NMOSD patients. Supplementary Table S3. Multivariable linear regression of the COMPASS-31 score in NMOSD patients. [file 12883_2023_3162_MOESM1_ESM.docx]

**Supplementary Table S1. The comparisons of COMPASS-31 score/subscore among NMOSD subgroups distributed by gender, serum AQP4-IgG positive or negative, clinical phenotype, and current preventive therapy.**

| NMOSD subgroup | COMPASS-31 total score, median [SD] (range) | *P* | orthostatic intolerance, median [SD] (range) | *P* | Vasomotor, median [SD] (range) | *P* | secretomotor, median [SD] (range) | *P* | gastrointestinal, median [SD] (range) | *P* | Bladder, median [SD] (range) | *P* | Pupillomotor, median [SD] (range) | *P* |
| --- | --- | --- | --- | --- | --- | --- | --- | --- | --- | --- | --- | --- | --- | --- |
| **Gender** |  | 0.21 |  | 0.99 |  | 0.06 |  | 0.19 |  | **0.02** |  | 0.71 |  | 0.83 |
| Female | 18.1 [10.7] (1-43) |  | 1.9 [2.4] (0-10) |  | 1.0 [1.8] (0-6) |  | 2.3 [1.7] (0-6) |  | 6.8 [4.1] (0-15) |  | 1.4 [1.7] (0-6) |  | 4.8 [3.6] (0-14) |  |
| Male | 13.8 [7.6] (5-30) |  | 1.5 [1.5] (0-4) |  | 0 (0) |  | 1.5 [1.6] (0-4) |  | 3.9 [3.0] (0-9) |  | 1.3 [1.9] (0-6) |  | 4.5 [3.3] (0-9) |  |
| **AQP4 IgG** |  | 0.88 |  | 0.81 |  | 0.84 |  | 0.99 |  | 0.64 |  | 0.34 |  | 0.72 |
| seropositivity | 17.2 [10.2] (1-43) |  | 1.8 [2.3] (0-10) |  | 0.8 [1.7] (0-6) |  | 2.1 [1.8] (0-6) |  | 6.1 [4.1] (0-15) |  | 1.3 [1.6] (0-6) |  | 4.8 [3.4] (0-14) |  |
| seronegativity | 17.3 [11.4] (6-34) |  | 2.0 [2.4] (0-7) |  | 1.0 [1.9] (0-5) |  | 2.0 [1.1] (1-4) |  | 6.6 [3.6] (2-11) |  | 2.1 [2.4] (0-6) |  | 4.4 [4.5] (0-11) |  |
| **Phenotype** |  | **0.03** |  | 0.50 |  | 0.18 |  | 0.19 |  | 0.73 |  | 0.35 |  | 0.13 |
| ON | 15.3 [9.1] (1-34) |  | 1.8 [2.9] (0-8) |  | 1.2 [1.9] (0-5) |  | 2.3 [1.5] (0-5) |  | 5.8 [4.8] (0-15) |  | 1.1 [1.5] (0-5) |  | 4.7 [3.3] (0-11) |  |
| TM | 14.1 [10.1] (1-43) |  | 1.8 [2.1] (0-7) |  | 0.3 [0.9] (0-4) |  | 1.6 [1.6] (0-5) |  | 6.0 [3.8] (0-14) |  | 1.2 [1.8] (0-6) |  | 3.7 [3.7] (0-14) |  |
| ON+TM | 21.0 [10.1] (5-41) |  | 1.8 [2.1] (0-10) |  | 1.0 [2.0] (0-6) |  | 2.5 [1.8] (0-6) |  | 6.6 [3.9] (0-12) |  | 1.7 [1.8] (0-6) |  | 5.6 [3.5] (0-11) |  |
| **Therapy** |  | 0.34 |  | 0.84 |  | **0.04** |  | 0.81 |  | 0.40 |  | 0.37 |  | 0.55 |
| MMF/AZA | 16.1 [10.3] (1-43) |  | 1.5 [1.7] (0-7) |  | 0.5 [1.4] (0-6) |  | 5.7 [3.7] (0-13) |  | 1.5 [1.8] (0-6) |  | 4.5 [3.8] (0-14) |  | 19.1 [9.6] (6-40) |  |
| RTX | 19.1 [9.6] (6-40) |  | 2.5 [3.3] (0-10) |  | 1.6 [2.1] (0-5) |  | 2.3 [1.6] (0-5) |  | 7.4 [4.7] (0-15) |  | 1.5 [1.8] (0-6) |  | 5.2 [2.9] (0-9) |  |
| other | 21.3 [2.9] (1-30) |  | 2.0 [1.8] (0-4) |  | 0 |  | 2.0 [2.3] (0-4) |  | 6.5 [2.3] (0-4) |  | 0.3 [0.5] (0-1) |  | 5.3 [3.9] (0-9) |  |

AQP4-IgG, IgG autoantibodies to aquaporin 4; ON, optica neuritis; TM, transverse myelitis; MMF, mycophenolate mofetil; AZA, azathioprine; RTX, rituximab; COMPASS-31, Composite Autonomic Symptom Score 31.

**Supplementary Table S2. The correlation between COMPASS-31 score/subscore and clinical variables in NMOSD patients.**

| Variables | COMPASS-31 total score | | Orthostatic Intolerance | | Vasomotor | | Secretomotor | | Gastrointestinal | | Bladder | | Pupillomotor | |
| --- | --- | --- | --- | --- | --- | --- | --- | --- | --- | --- | --- | --- | --- | --- |
|  | r | *P* | r | *P* | r | *P* | r | *P* | r | *P* | r | *P* | r | *P* |
| Age | 0.05 | 0.67 | -0.19 | 0.13 | -0.09 | 0.48 | 0.04 | 0.74 | -0.12 | 0.35 | -0.07 | 0.59 | 0.04 | 0.75 |
| BMI | -0.20 | 0.12 | 0.08 | 0.55 | 0.07 | 0.56 | **-0.26** | **0.04** | -0.09 | 0.47 | **-0.27** | **0.03** | -0.08 | 0.55 |
| Number of attacks | **0.49** | **<0.001** | 0.04 | 0.76 | 0.18 | 0.16 | 0.25 | 0.05 | 0.21 | 0.09 | 0.20 | 0.13 | 0.069 | 0.59 |
| Disease duration | **0.52** | **<0.001** | 0.15 | 0.24 | 0.20 | 0.12 | **0.27** | **0.03** | **0.29** | **0.02** | 0.18 | 0.16 | -0.01 | 0.96 |
| EDSS | **0.50** | **<0.001** | -0.02 | 0.86 | 0.07 | 0.61 | 0.24 | 0.06 | 0.16 | 0.20 | 0.24 | 0.06 | 0.11 | 0.39 |
| Cervical cord lesions | 0.23 | 0.07 | **0.29** | **0.02** | 0.09 | 0.46 | 0.15 | 0.23 | 0.05 | 0.68 | 0.17 | 0.19 | 0.10 | 0.46 |
| Thoracic cord lesions | **0.29** | **0.02** | -0.23 | 0.07 | 0.04 | 0.78 | 0.05 | 0.72 | 0.09 | 0.47 | 0.17 | 0.19 | 0.04 | 0.77 |
| Total number of spinal cord lesions | **0.35** | **0.005** | -0.02 | 0.88 | 0.07 | 0.59 | 0.14 | 0.28 | 0.07 | 0.59 | 0.24 | 0.06 | 0.13 | 0.29 |
| Anxiety (HADS-A) | **0.55** | **<0.001** | 0.13 | 0.31 | **0.35** | **0.004** | 0.14 | 0.28 | 0.10 | 0.44 | 0.10 | 0.45 | 0.03 | 0.83 |
| Depression (HADS-D) | **0.48** | **<0.001** | 0.08 | 0.53 | 0.20 | 0.13 | 0.15 | 0.25 | 0.19 | 0.13 | 0.05 | 0.72 | -0.03 | 0.83 |
| Sleep (PSQI) | **0.59** | **<0.001** | 0.03 | 0.79 | 0.07 | 0.60 | **0.30** | **0.02** | 0.21 | 0.11 | 0.16 | 0.21 | 0.20 | 0.11 |
| Fatigue (FSS) | **0.56** | **<0.001** | 0.15 | 0.24 | 0.19 | 0.14 | 0.11 | 0.39 | 0.13 | 0.33 | 0.23 | 0.07 | 0.01 | 0.94 |

COMPASS-31, Composite Autonomic Symptom Score 31; r, Spearman's ranked correlation; BMI, Body Mass Index; EDSS, Expanded Disability Status Scale; HADS, Hospital Anxiety and Depression Scale; PSQI, Pittsburgh Sleep Quality Index; BFI, Brief Fatigue Inventory; FSS, Fatigue Severity Scale.

**Supplementary Table S3.** **Multivariable linear regression of the COMPASS-31 score in NMOSD patients.**

| Variables | B | S.E. | 95%CI | *P* |
| --- | --- | --- | --- | --- |
| EDSS | 1.83 | 0.56 | 0.71 to 2.95 | 0.002 |
| Total number of spinal cord lesions | 0.59 | 0.22 | 0.15 to 1.02 | 0.009 |
| Fatigue (FSS) | 0.16 | 0.07 | 0.02 to 0.31 | 0.031 |

COMPASS-31, Composite Autonomic Symptom Score 31; B, beta-co-efficiency; S.E., standard error; EDSS, Expanded Disability Status Scale; FSS, Fatigue Severity Scale.
